# Magnesium, Aging, and the Elderly Patient

**DOI:** 10.1100/tsw.2004.114

**Published:** 2004-07-29

**Authors:** Mladen Davidovic, Dejan Trailov, Dragoslav Milosevic, Branimir Radosavljevic, Pavle Milanovic, Snezana Djurica, Helena Loncar-Stevanovic, Radmila Stevic

**Affiliations:** ^1^ KBC Zvezdara, Center of Geriatric Medicine, Beograd, Serbia and Montenegro; ^2^ Medical Faculty, Institute of Chemistry, Beograd, Serbia and Montenegro; ^3^ Medical Faculty, Institute of Physiology, Beograd, Serbia and Montenegro; ^4^ KBC Zvezdara,Center for radio-isotopes,Beograd, Serbia and Montenegro

**Keywords:** aging, magnesium, diuretics, elderly, oldest

## Abstract

Magnesium, beyond any doubt, plays an important role in metabolism. Alterations of magnesium levels have an impact on many organs and systems, especially during aging. We had 156 participants aged 60–93 years (average 74.7 years) in our survey. Of them, 49 were men and 107 were women. Treatment with loop diuretics (Furosemid and Bumetanide) and magnesium levels was correlated, as well as the influence of magnesium levels on life span. Serum magnesium levels were measured in patients receiving diuretics and in the control group. Also, magnesium levels were measured in patients who passed away in the course of their disease and were compared with the control group. Magnesium levels in the diuretic group (100 patients) were 0.93 ± 0.094 mmol/l, while the average levels in the control group of 56 patients were 0.89 ± 0.075 mmol/l. In 29 patients who passed away, average magnesium levels were 0.92 ± 0.078 mmol/l, while in the control group (127 patients), magnesium levels were 0.93 ± 0.083 mmol/l. The differences were not statistically significant. There were no differences in serum magnesium of the elderly persons investigated regarding age group, gender, or type of diuretics. If methods of determining ionizing magnesium in serum or intracellular magnesium are not available, normal magnesium values in the serum are to be taken with a qualified acceptance.

## INTRODUCTION

Aging is a universal process that results from the interaction of genomes and the environment. The organism is permanently affected by both environmental factors and intra- and intercellular adverse effects (free radicals, radiation, toxins, microorganisms, etc.)[1].

A growing number of publications stress magnesium as a clinically important electrolyte. Numerous clinical studies and case reports document a growing interest for magnesium as a therapeutic agent in coronary disease, arrhythmias, arterial hypertension, and asthma.

The range of magnesium in the elderly can be in disorder for two reasons: a deficit of magnesium or changes of metabolism of magnesium (the loss of magnesium). According to various authors, oxidative damage plays an important role in the process of aging. The tissue of animals with a deficit of magnesium are more sensitive to oxidative damage and peroxidation[2].

Hospitalized elderly people are especially sensitive. The main reasons are the less absorption of magnesium in the elderly (people of 70 years 30% less than people of 30 years), disordered metabolism of magnesium in bones, sometimes urinary leakage, less adaptability to stress, insulin resistance, and adrenaline hyperactivity. Secondary loss is connected with associated illnesses (diabetes, drugs, etc.)[3].

Based on the some recent studies, magnesium deficiency can influence the aging process in many different ways:

- Genetic stability plays an important role in individual longevity. Magnesium deficiency can cause inadequate DNA repair and lead to genetic instability, accelerated aging, and many diseases[4].
- Magnesium deficiency influences different cell functions and ends with senescence[5].
- Magnesium supplements can prolong the life span of rats[6].

From the clinical point of view it is very important to underline that:

- Potassium depletion is often (from 38–42%) associated with unrecognized magnesium deficiency[7].
- There is some proof that therapy with magnesium can act as an antiarrhythmic agent also in patients without magnesium depletion[8].

## MATERIALS AND METHOD

Our data showed that normal blood levels of magnesium, measured calorimetrically using Mann-Yoe method, range 0.89–0.94 mmol/l.

A consensus has not been established yet about normal serum levels of magnesium. There are no significant differences in magnesium levels among different age groups[9], but circadian variations do exist, as well as gender variations in healthy persons[10]. Published studies revealed different values, ranging from 0.79 ± 0.06 mmol/l[11], through 0.81 ± 0.08 mmol/l[11] and 0.82 ± 0.07 mmol/l[10] to 0.83 ±0.07 mmol/l[12].

## RESULTS

We had 156 participants aged 60–93 years (average 74.7 years) in our survey. Of them, 49 were men and 107 were women. Treatment with loop diuretics (Furosemid and Bumetanide) and magnesium levels was correlated, as well as the influence of magnesium levels on life span.

Serum magnesium levels were measured in patients receiving diuretics and in the control group. Also, magnesium levels were measured in patients who passed away in the course of their disease and were compared with the control group.

There were no differences in serum magnesium of the elderly persons investigated regarding age group, gender, or type of diuretics (Bumetanide or Furosemid).

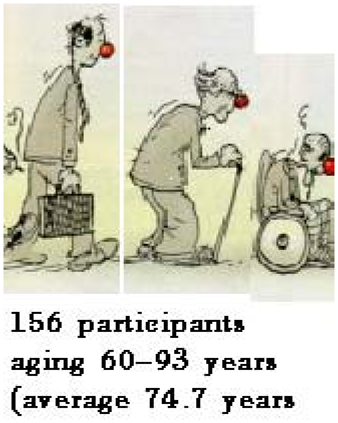

**Fig. 1** Investigated persons

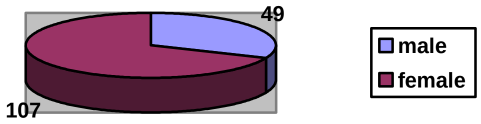

**Fig. 2** Sex ratio

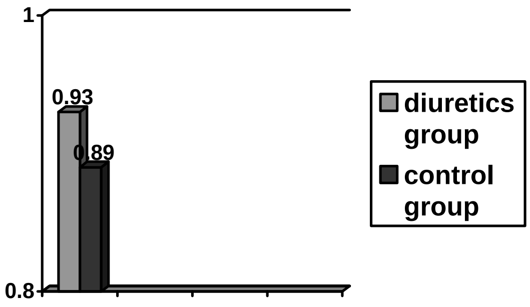

**Fig. 3** Serum magnesium levels in patients receiving diuretics and control group

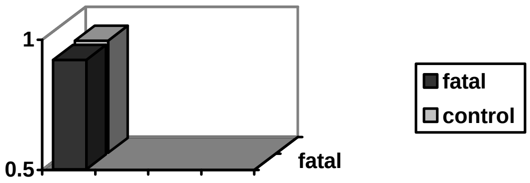

**Fig. 4** Serum magnesium levels in lethal and non-lethal group

In 29 patients who passed away, average magnesium levels were 0.92 ± 0.078 mmol/l, while in the control group (127 patients), magnesium levels were 0.93 ± 0.083 mmol/l. The differences were not statistically significant.

## DISCUSSION

Some experimental results have demonstrated the constancy of magnesium status throughout the adult lifetime[14]. There were no significant differences in total magnesium concentration of various tissues (plasma, erythrocytes, brain, testicles, heart, skeletal muscle, gastrointestinal tract, kidney, femoral bone) in Wistar male rats of different ages. The oldest group of rats tended to decreased intestinal magnesium absorption.

In order to clarify if the need for magnesium increased with aging, two age-different rats groups were orally supplemented with two magnesium acetate solutions and mineral water rich with magnesium during several weeks[15,16,17]. The magnesium supplementation did not affect magnesium content in any rat tissue, as well as proteins and high-density lipoproteins concentrations in serum.

On the other hand, the beneficial effect of magnesium supplementation on serum cholesterol concentration is noticed, expressed more in younger animals, and on triglycerides, observed only in younger ones again, suggesting that older animals needed increased amounts of magnesium compared to younger rats to attain the similar consequence.

Although the results are in accordance with some literature data, having in mind to establish more exactly the effect of rats aging on magnesium contents and distribution in tissues as well as on possible pharmacological magnesium potency, it is necessary to experiment with older rats that are more age different, to use other supplements of higher magnesium concentrations during longer periods and finally to determine any other biochemical constituents.

It is well known that it is impossible to determine the exact magnesium status of some individuals because it is a sum of intra- and extracellular concentrations of ionized magnesium and magnesium bound to proteins and inorganic ligands. Consequently, it is imperative to determine not only the total magnesium concentration, as it has been done in the presented references by using the reference method of atomic absorption spectrophotometry, but also to analyze the free ionized intra- and extracellular magnesium, the only biologically and physiologically active forms.

In humans, it is preferred that a time-consuming tolerance test (recommended by the International Federation of Clinical Chemistry) be performed simultaneously with a determination of ionized and total magnesium concentration in serum or plasma and intracellular magnesium in blood cells.

Other studies presented similar results. After treatment with hydrochlorothiazide, plasma levels of magnesium did not change, although erythrocyte magnesium levels decreased[13]. Hypomagnesiaemia related with loop diuretics can be associated with muscle spasms and tetany resistant to supplementation of potassium and calcium[18]. Magnesium supplementation is absolutely indicated in all patients taking thiazide or loop diuretics on a long-term basis, and it is of essential importance to measure levels of intracellular magnesium, since plasma levels can be falsely normal[11]. Correlation between magnesium levels in erythrocytes and severity of myocardial infarction has been found[19]. Low magnesium levels are not associated with the increased risk of ventricular fibrillation[20]. Physiological supplementation of magnesium in doses of 15 mmol/day had no influence on serum levels, left ventricular ejection fraction, and overall prognosis in patients with primary dilated cardiomyopathy.

In the course of various illnesses, the level rise of magnesium can be seen as well as normal values, or risen values. It seems that the pull of magnesium, better to say its metabolism, is much more important than the exact values itself[12].

Nowadays, more and more scientists are interested in the connection between magnesium and hypertension. Experimental research showed that lack of magnesium in food is connected with a predisposition to hypertension. Some clinical studies showed the connection between an antihypertension effect and increased intake of magnesium.

The aim of this study[20] was to investigate the connection of taking salt with metabolism magnesium in patients with hypertension.

There were 50 patients within the study without secondary causes of hypertension and those who suspended antihypertension therapy 2 weeks before the research began. Stable hypertension was considered to be the report of constantly high blood pressure more than 160/90 mmHg without therapy that did not fall to normal after 4 days of resting. Patients were at a higher, lower, and normal regime of taking salt (20 mMol NaCl and 200 mMol NaCl daily). This group of patients was divided between salt-sensitive and salt-insensitive according to changes of medium anterior pressure during the various regimes of taking salt by food.

The average values of magnesium in serum at all regimes of salt intake were within normal standards. Statistically less value of magnesium in serum was found in salt-sensitive patients during nonsalt nutrition. There were no other statistically important differences.

This study did not show any significant correlation between magnesium and blood pressure either to the whole group or for salt-sensitive patients. It could be seen that magnesium was a strong antagonist digitalis, in other words taking more magnesium could block the hypertension effect of taking more salt. It was found that hypertension effect of magnesium combined with higher concentration of magnesium in erythrocytes. These results are the same as a few experimental studies.

The results of this study brings out the conclusion that the value of serum magnesium is lower in salt-sensitive patients and that there are possibilities for a therapeutic effect of magnesium for these patients[21].

## CONCLUSION

Magnesium is an important electrolyte in other diseases. Numerous studies constantly prove that the insulin-independent diabetes mellitus is associated with hypomagnesiaemia. Also, hypomagnesiaemia is associated with the increased production of free oxygen radicals. Low levels of magnesium increase thrombocyte aggregability and adhesivity.

Magnesium therapy can be performed within supplementation (5 mg/kgTT) and those doses are completely safe. B6, vitamin D, and selenium are additionally given as help to this therapy. When diuretics are given and the level of magnesium is decreasing, the hydrochlorothiazides can be given if possible. Indications for intensive therapy with magnesium (>10 mg/kgTT) can be within various gynecological, cardiological, anesthesiological, toxicological, and other therapeutic procedures. In general, in geriatrics, the main importance is the cytoprotective role of magnesium, which could theoretically extend years of living[22].

One up-to-date noninvasive method of measuring magnesium is concerned with its level in epithelial cells sublingual mucous membrane. In those cells, the levels of magnesium are in correlation with the level in the cells of cardiac atria and little with serum level[23].

If we accept as a fact that a deficit of magnesium is essential in the disorder of the lipid status and development of atherosclerosis, it would play a significant role in early aging.

Serum magnesium, however, does not reflect the actual status because the mentioned disorder of the lipid status disrupts the real registered values of magnesium in the serum[24].

Based on the presented data, it could be concluded that average serum magnesium levels do not represent the real state.

## ADDITIONAL REFERENCES

* Tsutsui, M., Shimokawa, H., Yoshihara, S., et al. (1993) Intracellular magnesium deficiency in acute myocardial infarction. *Jpn. Heart J.* **34(4),** 391–401.
* Loncar-Stevanovic, H., Vujovic, Z., Potkonjak, B., Radosavljevic, B., and Jovanovic, T. (1999) The influence of magnesium supplementation on its content of several adult rats tissues. In *Magnesium in the Environment and in Organisms*. Maksimovic, Z.J., Gašic, M.J., and Spasic, M.B., Eds. Serbian Academy of Sciences and Arts Scientific Meetings.Volume XCII, Book 1. pp. 89–96.
* Rudnicki, M., Jorgenses, T., Jensen, K.H., et al. (1993) Calcium, magnesium, and free fatty acids in the formation of gallstones: a noted case-control study. *Am. J. Epidemiol*. **137(4),** 404–408.
* Tohno, S., Tohno, Y., Moriwake, Y., Azuma, C., Ohnishi, Y., and Minami, T. (2002) Compositional changes of the aortic valve similar to the artery with aging. *Biol. Trace Elem. Res.* **87(1–3),** 83–93.
* Swain, R. and Kaplan-Machlis, B. (1999) Magnesium for the next millennium. *South. Med. J.* **92(11)**, 1040–1047.
* Durlach, J., Bac, P., Durlach, V., Rayssiguier, Y., Bara, M., and Guiet-Bara, A. (1998) Magnesium status and ageing: an update. *Magnes. Res*. **11(1),** 25–42.
* Eaton, L. (2002) Magnesium could save hundreds of women's lives worldwide. *BMJ* **324**, 1351.
